# Tissue Tropisms and Transstadial Transmission of a *Rickettsia* Endosymbiont in the Highland Midge, Culicoides impunctatus (Diptera: Ceratopogonidae)

**DOI:** 10.1128/AEM.01492-20

**Published:** 2020-10-01

**Authors:** Jack Pilgrim, Stefanos Siozios, Matthew Baylis, Gregory D. D. Hurst

**Affiliations:** a Institute of Infection, Veterinary and Ecological Sciences, Faculty of Health and Life Sciences, University of Liverpool, Liverpool, United Kingdom; b Health Protection Research Unit in Emerging and Zoonotic Infections, Liverpool, United Kingdom; University of Bayreuth

**Keywords:** symbiosis, microbial ecology, *Rickettsia*, *Culicoides*, arthropod vectors, tissue tropisms, vector biology

## Abstract

*Rickettsia* is a genus of intracellular bacteria which can manipulate host reproduction and alter sensitivity to natural enemy attack in a diverse range of arthropods. The maintenance of *Rickettsia* endosymbionts in insect populations can be achieved through both vertical and horizontal transmission routes. For example, the presence of the symbiont in the follicle cells and salivary glands of *Bemisia* whiteflies allows Belli group *Rickettsia* transmission via the germ line and plants, respectively. However, the transmission routes of other *Rickettsia* bacteria, such as those in the Torix group of the genus, remain underexplored. Through fluorescence *in situ* hybridization (FISH) and transmission electron microscopy (TEM) screening, this study describes the pattern of Torix *Rickettsia* tissue tropisms in the highland midge, *Culicoides impunctatus* (Diptera: Ceratopogonidae). Of note is the high intensity of infection of the ovarian suspensory ligament, suggestive of a novel germ line targeting strategy. Additionally, localization of the symbiont in tissues of several developmental stages suggests transstadial transmission is a major route for ensuring maintenance of *Rickettsia* within *C. impunctatus* populations. Aside from providing insights into transmission strategies, the presence of *Rickettsia* bacteria in the fat body of larvae indicates potential host fitness and vector capacity impacts to be investigated in the future.

**IMPORTANCE** Microbial symbionts of disease vectors have garnered recent attention due to their ability to alter vectorial capacity. Their consideration as a means of arbovirus control depends on symbiont vertical transmission, which leads to spread of the bacteria through a population. Previous work has identified a *Rickettsia* symbiont present in several species of biting midges (*Culicoides* spp.), which transmit bluetongue and Schmallenberg arboviruses. However, symbiont transmission strategies and host effects remain underexplored. In this study, we describe the presence of *Rickettsia* in the ovarian suspensory ligament of *Culicoides impunctatus*. Infection of this organ suggests the connective tissue surrounding developing eggs is important for ensuring vertical transmission of the symbiont in midges and possibly other insects. Additionally, our results indicate *Rickettsia* localization in the fat body of *Culicoides impunctatus*. As the arboviruses spread by midges often replicate in the fat body, this location implies possible symbiont-virus interactions to be further investigated.

## Abstract (precis)

Microbial symbionts of disease vectors have garnered recent attention due to their ability to alter vectorial capacity. Their consideration as a means of arbovirus control depends on symbiont vertical transmission, which leads to spread of the bacteria through a population. Previous work has identified a *Rickettsia* symbiont present in several species of biting midges (*Culicoides* spp.), which transmit bluetongue and Schmallenberg arboviruses. However, symbiont transmission strategies and host effects remain underexplored. In this study, we describe the presence of *Rickettsia* in the ovarian suspensory ligament of *Culicoides impunctatus*. Infection of this organ suggests the connective tissue surrounding developing eggs is important for ensuring vertical transmission of the symbiont in midges and possibly other insects. Additionally, our results indicate *Rickettsia* localization in the fat body of *Culicoides impunctatus*. As the arboviruses spread by midges often replicate in the fat body, this location implies possible symbiont-virus interactions to be further investigated.

## INTRODUCTION

Heritable microbes of arthropods are important drivers of diverse host phenotypes. For example, both *Rickettsia* and *Wolbachia* are associated with reproductive parasitisms which favor the production of female offspring (e.g., male-killing and parthenogenesis) (1–3), while also being associated with resistance or tolerance against pathogens (4–6). Specifically, in disease vectors such as mosquitoes, both naturally occurring and artificially introduced symbionts can lead to a “virus-blocking” effect (7–10). These phenotypes, combined with maternal inheritance, drive the symbiont (and its effects on vectorial capacity) into a population and are currently being considered as a means of arbovirus control (11–13).

*Culicoides* biting midges (Diptera: Ceratopogonidae) are vectors which transmit economically important pathogens of livestock, including bluetongue and Schmallenberg viruses (14). Currently, disease control primarily relies on vaccines which, given the rapid emergence and spread of these viruses, are often not available. Thus, alternate strategies, such as those based on symbionts, are of particular interest for midge-borne pathogens. So far, three endosymbionts have been observed in *Culicoides* spp.: *Wolbachia*, *Cardinium*, and *Rickettsia* (15–18). Of these, symbioses with *Rickettsia* are the most common, and are present in several midge vector species (17). However, effects of the *Rickettsia* on the host are yet to be determined. The absence of sex-ratio distortion suggests the lack of a reproductive parasitism, and as some midge populations do not carry *Rickettsia* at fixation (excluding an obligate association), this indicates that the drive of this endosymbiont might be related to a facultative benefit (e.g., pathogen protection).

The highland midge, *Culicoides impunctatus*, is a biting nuisance in west Scotland, with “midge attacks” accountable for significant economic impact through losses in tourist and forestry industries (19, 20). Their ability to reproduce once in the absence of a blood meal (autogeny) means that huge numbers can develop even where vertebrate hosts are not available (21–23). Thus, it is interesting to speculate that *Rickettsia* may play a possible role in autogeny. Indeed, Rickettsia felis is necessary for egg development in the booklouse Liposcelis bostrychophila (24). As autogeny is responsible for the pest burden of *C. impunctatus*, this could offer a target for population suppression in the future.

This study focuses on broadening our understanding of the interactions between *C. impunctatus* and its symbiont, a Torix group *Rickettsia* strain. The presence of *Rickettsia* in *C. impunctatus* oocytes has previously been identified and indicates transovarial transmission (17). However, the route of migration to egg chambers by the symbiont is not clear and the tropisms to other tissues remain unexplored. Vertical transmission of nonobligate symbionts is achieved through diverse modes of germ line targeting (25). With certain *Wolbachia* strains of *Drosophila* spp., the symbiont localizes in the germ line stem cell niche continuously throughout development (26–28). For other symbionts, such as Belli and Adalia group *Rickettsia* spp., germ line localization follows infection of somatic tissues associated with the germ line (e.g., follicle cells and bacteriocytes) (29–31). Intriguingly, *Rickettsia* also has the unusual ability to infect sperm head nuclei, allowing for paternal inheritance, which can combine with maternal transmission to drive a costly symbiont into the population (32). We therefore aimed to explore *Rickettsia* localization in the midge germ line and its associated tissues to glean insights into germ line targeting mechanisms in this symbiosis.

Another objective of this project was to generate hypotheses of symbiont function through examining patterns of somatic tissue localization. For instance, the close association of *Blattabacterium* with uric acid-containing cells (urocytes) in cockroaches is associated with nitrogen recycling into amino acids (33, 34), while the presence of *Wolbachia* in specific areas of the *Drosophila* brain is linked to mate choice (35). Furthermore, horizontal transmission pathways can be elucidated in a similar manner, with *Rickettsia* salivary gland infections of haematophagous and phloem-feeding arthropods reflecting transmission to vertebrates and plants, respectively (30, 36, 37). In light of this, through fluorescence *in situ* hybridization (FISH) and transmission electron microscopy (TEM) screening, this study describes patterns of *Rickettsia* infection in both germ line and somatic tissues in multiple developmental stages of *C. impunctatus*.

## RESULTS

### *Rickettsia* infection during oogenesis.

**FIG 1:**
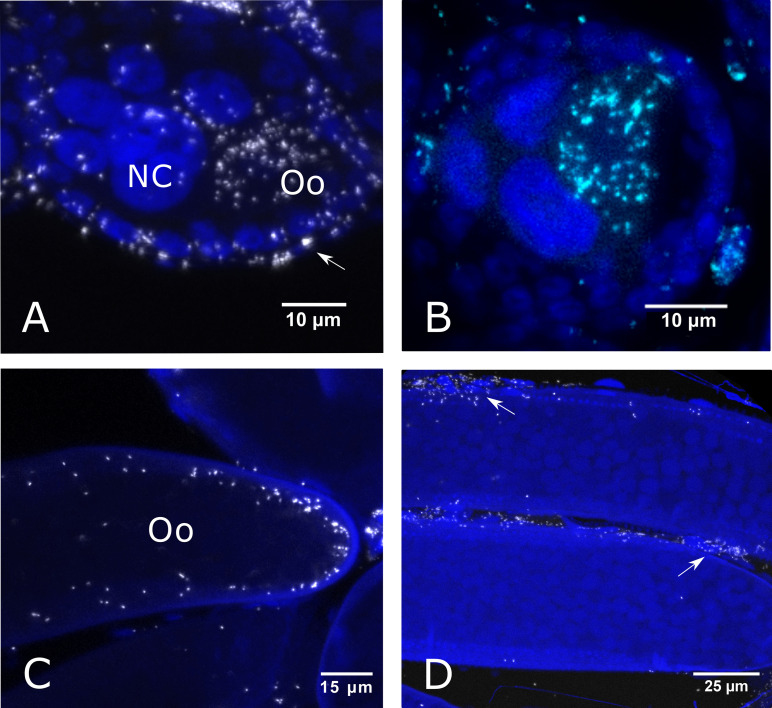
FISH images of *C. impunctatus* egg chambers at different developmental stages of oogenesis. *Rickettsia*-specific probe (white); DAPI staining (blue). (A) *Rickettsia* infection of stage 1 eggs (0 h post-blood feeding) with predominant localization in the oocyte (Oo), nurse cells (NC), and follicle cells (arrow). (B) *Rickettsia* infection of stage 2 eggs (12 h post-blood feeding) with the accumulation of cloudy yolk deposits in the oocyte (Oo). Infection is still primarily observed in the oocyte (Oo), although infection of nurse cells (NC) is now absent. (C) A focal plane of stage 4 eggs (120 h post-blood feeding) showing localization at the periphery of the oocyte (Oo). (D) A focal plane of stage 4 eggs (120 h post-blood feeding) showing infection of follicle cells (arrows).

**FIG 2:**
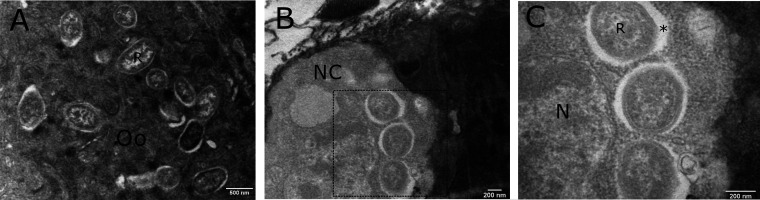
Transmission electron microscopy (TEM) images of *C. impunctatus*-infected stage 1 egg chambers. (A) TEM section of a *C. impunctatus* egg demonstrating *Rickettsia* clusters in the oocyte cytoplasm (Oo). (B) TEM section of a *C. impunctatus* egg demonstrating the presence of *Rickettsia* bacteria in a nurse cell (NC). (C) Magnified details of the box in panel B, demonstrating perinuclear *Rickettsia* (R). *, radiolucent halo/slime layer; N, nucleus.

Early stage (stage 1) egg chambers contained *Rickettsia* clusters predominantly in oocytes in which there was no presence of yolk deposition observed (Fig. 1A and 2A). The signal was strongest in the oocyte, with bacteria also being observed within nurse cells. Electron microscopy images suggested that the *Rickettsia* bacteria are perinuclear rather than within the nurse cell nuclei themselves (Fig. 2B and C). The follicular epithelium was also infected with bacteria seen in transit between follicle cells and the oocyte (Fig. 1A). After 48 h post-blood feeding, yolk deposition was seen as a clouding in the (stage 2) oocyte, although individual yolk granules were not visible (Fig. 1B). Clusters of bacteria no longer occupied nurse cells but still predominantly filled the oocyte. In stage 4 eggs, yolk granules became visible and appeared to harbor sparse bacteria with a predominant localization in follicle cells (Fig. 1D). However, in certain focal planes, *Rickettsia* bacteria were observed in the oocyte cytoplasm but present only at the periphery of the oocyte (Fig. 1C).

### *Rickettsia* localization to the ovarian suspensory ligament.

**FIG 3:**
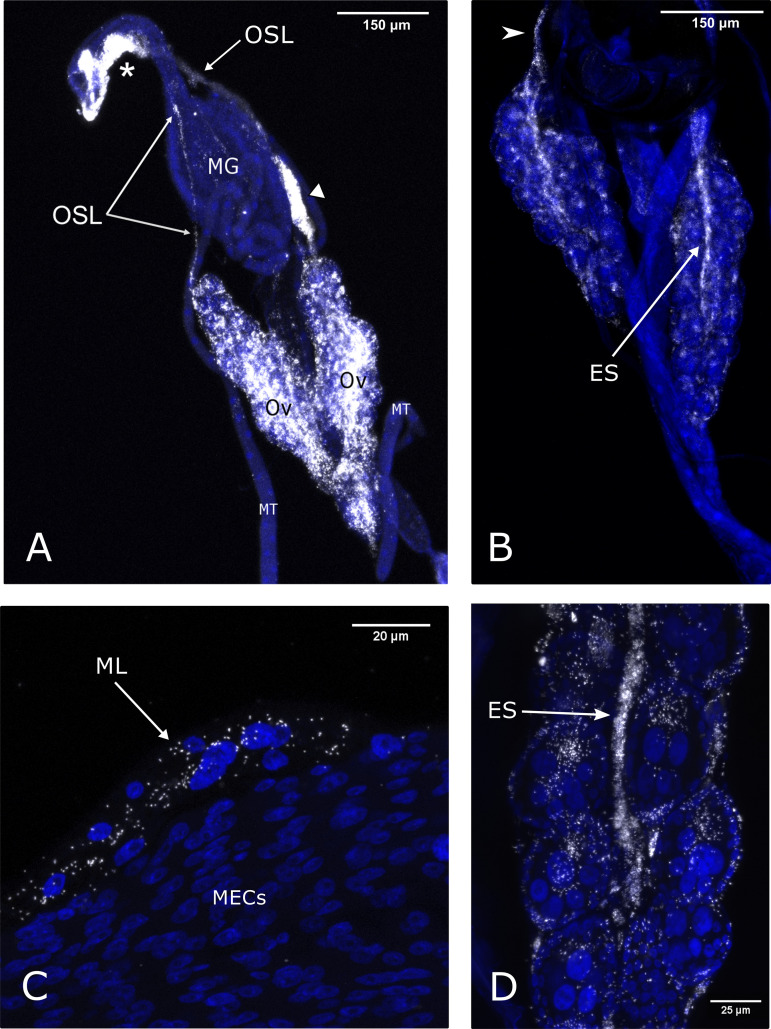
*Rickettsia* localization in *C. impunctatus* adult connective tissues associated with both the midgut and ovaries via FISH imaging. *Rickettsia*-specific probe (white); DAPI staining (blue). (A) Strong *Rickettsia* signals identified at the anterior-posterior midgut junction (*) as well as the paired ovaries (Ov). These two areas are connected via the ovarian suspensory ligament (OSL) which runs from the midgut junction to the apex of the ovary. White triangle, putative fat body lobe; MG, midgut; MT, Malpighian tubules. (B) A focal plane of the paired ovaries demonstrating the continuation of the suspensory ligament attachment site at the ovary apex (arrowhead) into the ovary. (C) *Rickettsia* localization at the median ligament (ML) and the fusion of the ovarian suspensory ligaments at the attachment site at the anterior-midgut junction. Lack of infection is observed in midgut epithelial cells (MECs). (D) The continuation of the ovarian suspensory ligament with the ovarian epithelial sheath (ES) allows for the delivery of *Rickettsia* into neighboring egg chambers.

Initial examination of adult specimens at low magnification gave a consistent pattern of strong localized signal at the anterior/posterior midgut junction (Fig. 3A). On examination at a higher magnification, no signal was observed in any of the midgut epithelial cells (Fig. 3C). Further scrutiny of individuals led to the discovery that the infected structure was the ovarian suspensory ligament. This structure, otherwise known as the “median ligament,” was seen to pair off and loop down from the midgut attachment site before attaching at the apex of the ovary (Fig. 3A to C). It was possible to follow the signal down the suspensory ligament where the structure became continuous with the terminal filaments of ovarioles and the ovarian epithelial sheath (Fig. 3B and D), the structure encasing ovarioles. Strength of infection was consistent in the ovarian epithelial sheath over the long axis of the ovary, with neighboring immature egg chambers seen to be heavily infected. Bacteria could be seen migrating from this densely populated structure into neighboring follicle cells and further into oocytes themselves (Fig. 3D). Within the ovary, the germarium appeared to be no more strongly infected than the rest of the ovarian tissue. In one individual, the attachment of the suspensory ligament to a sparsely cellular but strongly signaled structure is thought to be part of a lobe of the fat body (Fig. 3A), although the ethanol-based fixative (Carnoy’s) diminishes lipids, leading to ambiguity of identification when observed under transmitted light.

### *Rickettsia* infection in other adult tissues.

**FIG 4:**
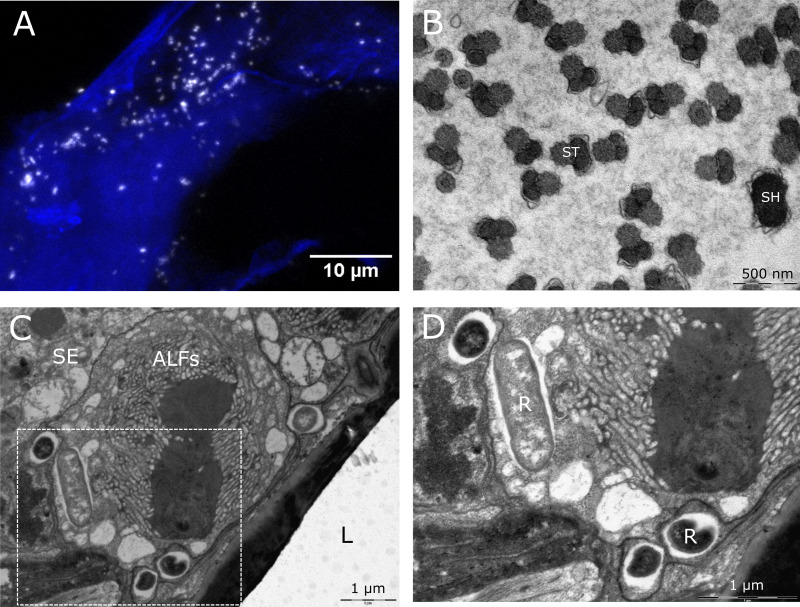
FISH and TEM analysis of *C. impunctatus* spermathecae. (A) FISH image of *Rickettsia* bacteria in a crushed spermatheca. *Rickettsia*-specific probe (white); DAPI staining (blue). (B) TEM section of *Rickettsia*-uninfected sperm heads (SH) and sperm tails (ST). (C) TEM section of junction between the spermathecal lumen (L) and spermathecal epithelium (SE). ALFs, actin-like filaments. (D) Higher magnification details of the box in panel C, demonstrating longitudinal and cross-sectioned *Rickettsia* (R) bacteria residing in the spermathecal epithelium.

In the single male specimen available for analysis, infection of the testes was observed (Fig. S1B in the supplemental material). *Rickettsia* was further detected in crushed spermathecae from fertilized females (Fig. 4A). Subsequently, TEM sections of spermathecae were assessed to clarify the nature of this signal. Overall, infected spermathecae showed no evidence of bacteria in sperm heads or tails of spermatids or in the acellular matrix (Fig. 4B). However, *Rickettsia* was identified in the maternally derived spermathecal epithelium (Fig. 4C and D). Unfortunately, due to the difficulties of laboratory maintenance of *Culicoides*, a crossing system to definitively rule out paternal transmission was not possible. Finally, a single infection was observed in the crop of the foregut (Fig. S1A), but no signal was observed in Malpighian tubules, heads, or salivary glands for any adult sample.

### Subcellular location and associations.

Transmission electron microscopy of spermathecae and immature eggs revealed coccobacilli presumed to be *Rickettsia* in the cytoplasm of oocytes and spermathecal epithelial cells (Fig. 2A; Fig. 4C to D). Sections of bacteria ranged up to 1.35 μm in length and were seen together either in clumps, likely a result of recent division, or diffusely in tissues. The ultrastructure of *Rickettsia* demonstrated a distinctive slime layer/radiolucent halo typical of the genus (Fig. 2C). Infection was not observed in host cell nuclei.

### Larval tissue localization.

**FIG 5:**
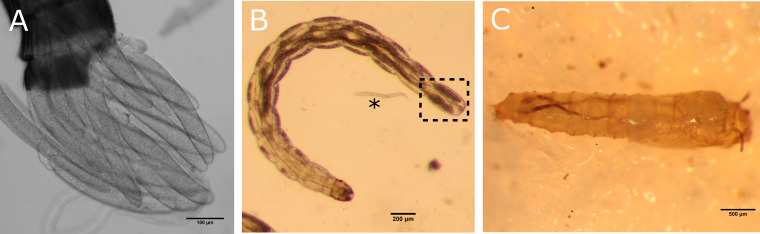
Transmitted light microscope images of different life stages of *C. impunctatus*. (A) Stage 4 eggs. (B) L3 larva with lateral fat bodies terminating in the terminal abdominal segment (box); *, *Panagrellus nephenticola* nematode. (C) Pupa.

**FIG 6:**
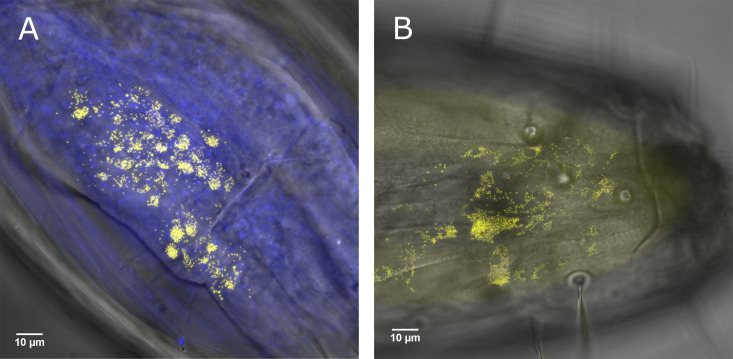
FISH imaging analysis of an L3 *C. impunctatus* larva. *Rickettsia*-specific probe (yellow); DAPI staining (blue). (A) Fat body *Rickettsia* infection of the terminal abdominal segment. (B) *Rickettsia* larval head infection.

Out of ten L3 larvae of unknown infection status, four showed a positive signal in the terminal abdominal (anal) segment fat body (Fig. 5B; Fig. 6A), with three of these also demonstrating infection in the heads (Fig. 6B). One individual of the four positives showed sporadic multifocal infections across the rest of the length of the body. Focal “burst” patterns of signal in the terminal abdominal segment suggest infections of globe-like structures, such as cells or lipid droplets of the fat body. Infections in the head could not be localized to the exact tissue infected, but *Rickettsia* was closely associated with the head body wall (within 0 to 3 μm proximity of the autofluorescent cuticle of each focal plane). Concurrent presence in the large fat-bodied anal segment of the larvae, alongside the fat body’s frequent attachment to the body wall, suggests the pericerebral fat body is the most likely tissue to be infected, although further work is needed to confirm this.

## DISCUSSION

*Rickettsia* bacteria are important components of arthropod biology, contributing to host protection against pathogens (5, 6) while also being causative agents of disease in their own right (38). The transmission routes of *Rickettsia* endosymbionts in biting midges are underexplored but can be informed by symbiont tissue localization. Although the maternal transmission of obligate symbionts is generally dependent on specialized cells (bacteriocytes), facultative (secondary) symbionts utilize various means to target the germ line (25). These can come in the form of cooption of yolk granules to gain entry into egg chambers via endocytosis (e.g., *Spiroplasma* in Drosophila melanogaster) (39), or through the continuous association with the germ line during morphogenesis (e.g., *Wolbachia* in *Drosophila* spp.) (26, 28). These modes of germ line targeting can be dismissed in the case of *C. impunctatus-Rickettsia* symbioses. Oocyte symbiont infection precedes yolk deposition, indicating no route through cooption of the vitellogenin transport systems (Fig. 1A), and larval stages show no localization to the midabdominal area in which the germ line progenitors are present (infection is in the head and terminal segments) (Fig. 6). Intriguingly, our description of the *Rickettsia*-infected ovarian suspensory ligament in *C. impunctatus* indicates a potential novel means of endosymbiont germ line targeting (Fig. 3).

The continuation of the suspensory ligament with the ovarian epithelial sheath (Fig. 3B and D) has previously been described in insects (40), with our results suggesting these tissues act as an intermediary for ensuring *Rickettsia* infection of egg chambers via follicle cells. The passage of *Rickettsia* through the follicle cells of ovarioles occurs in the ladybird Adalia bipunctata and the whitefly Bemisia tabaci (29, 30). In these instances, it is possible that *Rickettsia* migrates through follicle cells after ovarian contact with infected hemocytes/hemolymph (30, 41). Other studies have suggested bacteriocytes could be a means of transovarial transmission for *Rickettsia* (31, 42, 43), although further investigation suggested this was not a primary route of ovary targeting in whiteflies (30). In light of this, it is conceivable that infection of connective tissue directly linking the germ line allows for the vertical transmission of *Rickettsia* in midges and other insects.

Our findings of *Rickettsia* infection in follicle cells, but with apparent limited infection of mature oocytes (Fig. 1C and D), corroborate previous observations in the whitefly Bemisia tabaci (30). In this case, heavy *Rickettsia* infection in immature oocytes, but not mature stages, was attributed to younger eggs being more permeable than their mature counterparts. Alternatively, it may be the case that this perceived density variation is the result of a dilution effect as the oocyte gets bigger. The *Rickettsia* infection and close contact of the ovarian epithelial sheath to the follicle cells offer a mechanism for ensuring persistent follicular infection (Fig. 3D). Furthermore, as only a few bacterial cells are required to ensure subsequent infection of life stages (32), remnant *Rickettsia* in the oocyte periphery of mature eggs (Fig. 1C) appears to be sufficient for transstadial transmission (Fig. 6).

Although intranuclear infections are rare for bacteria, various *Rickettsia* strains have been observed to reside within nuclei (32, 43–45) with no indication of a detrimental impact on arthropod reproduction or development. Of particular interest is a Torix group *Rickettsia* strain that undergoes paternal transmission via sperm head nuclei in the leafhopper Nephotettix cincticeps (32). The additive effects of maternal and paternal transmission not only solidify vertical transmission but can also drive symbiont evolution through lineage mixing in the absence of any benefit of infection. In the current study, initial localization of *Rickettsia* bacteria to testes and spermathecae (Fig. 4A; Fig. 1B) suggested that intrasperm transmission may also be occurring in *C. impunctatus*. However, TEM sections of spermathecae provided evidence only for *Rickettsia* infection of maternally derived epithelia, with no bacteria observed in sperm head nuclei. Similarly, nurse cells of ovaries demonstrated the presence of perinuclear *Rickettsia* only (Fig. 2C). The absence of an intranuclear tropism in eggs as well as sperm suggests the capacity to infect nuclei may be evolutionary labile.

While vertical transmission is the primary transmission route for endosymbionts, horizontal transfer frequently occurs in *Rickettsia* and has allowed for the infection of a diverse range of organisms, including protists, arthropods, plants, and vertebrates (46, 47). The lack of a *Rickettsia* infection signal in salivary glands of *C. impunctatus*, and the parity of prevalence in male and female midges (17), indicates horizontal transmission to vertebrate hosts via hematophagy is unlikely.

Another finding of interest is the localization of *Rickettsia* to the fat bodies of larvae (Fig. 6A). Previous examples of endosymbiont fat body infections of *Culicoides* include the observation of “Rickettsia-like” organisms by Hertig and Wolbach (48) and “twinkling” symbionts under polarized light by Lawson (49). The larval fat body largely comprises two bands extending down the lateral abdomen, terminating in large lobes of the terminal segment (49) (Fig. 5B). *Rickettsia* appears to be one of few known endosymbionts to reside in the fat body, along with *Blattabacterium* of cockroaches (34) and *Wolbachia* in a variety of insects (50–52). The presence of *Wolbachia* in the fat body, an important endocrine tissue, has been associated with effects on host glucose and glycogen metabolism via altered enzyme activity and insulin signaling (53, 54). Not only is the fat body metabolically active, allowing for the bacterial sequestering of metabolite precursors, but fat cells are refractive to degradation during larval and pupal development (55, 56). Thus, this tissue offers a suitable niche in arthropods for stable division and proliferation of endosymbionts.

Other *Rickettsia*-infected regions of note are larval head tissues (Fig. 6B). Although it is not clear exactly which tissues are affected by the *Rickettsia*, the pericerebral fat body and cerebral ganglia (brain) are two candidates for future consideration. Endosymbiont brain infections have been proposed to lead to behavioral modifications (35, 57). Additionally, the fat body surrounding the brain has been demonstrated to play a different physiological role to that in the abdomen. Namely, unique insulin signaling pathways have been observed in the head fat bodies of Drosophila melanogaster, leading to increased longevity as a result of inhibited senescence (58).

The presence of *Rickettsia* in the fat body of *Culicoides*, a vector of several veterinary viruses, raises questions on the effects of fat bodies on vector competence. Bluetongue virus (BTV) and epizootic haemorrhagic disease virus (EHDV) of ruminants replicate in the fat body of midges (59, 60) before travelling to the salivary glands, suggesting interactions between *Rickettsia* and the virus could be occurring. For example, competition for lipids between bacteria and virus has been suggested to influence viral titers (61–64). Additionally, antimicrobial peptides are synthesized in the fat body (65) and are active against arboviruses (66), again suggesting that *Rickettsia* effects on vectorial capacity warrant further investigation.

The role of *Rickettsia* in the egg development of booklice (24) suggests a possible link between Torix *Rickettsia* and autogeny in *C. impunctatus*. Unfortunately, the difficulty in maintaining *C. impunctatus* colonies is a hindrance to investigating such host effects. The rearing methods of this study allowed for development to pupae (Fig. 5C) but overall survival was poor, with none completing a full life cycle (Fig. S2). Thus, further optimization of *C. impunctatus* rearing is needed to investigate this symbiotic system further.

In conclusion, this study has identified several somatic and germ line infections of a Torix *Rickettsia* endosymbiont in several biting midge life stages. Infection of the ovarian suspensory ligament, a continuation of the ovarian epithelial sheath, has identified a potential novel means of endosymbiont germ line targeting. Additionally, a somatic tissue infection to study further is the fat body, which could have implications for host effects, as well as arbovirus transmission dynamics.

## MATERIALS AND METHODS

### *Culicoides impunctatus* collections.

*Culicoides impunctatus* collection for imaging of adult tissues was carried out at multiple sites in the United Kingdom: Dumfries, Kielder forest, Loch Lomond and Fort William between June 2017 and September 2018. Populations at these sites were caught by light traps and confirmed as *Rickettsia*-infected (at fixation) by PCR of the *gltA* gene as previously described (17). Sanger sequencing of amplicons confirmed the *Rickettsia* strain as belonging to the Torix group and identical to the *C. impunctatus* strain previously reported (KY765379).

For midges not used for ovipositing, individual *Culicoides* midges were allowed to rest on authors’ arms before aspiration into 1.5-ml Eppendorf tubes with 10% sucrose-soaked cotton wool placed at the bottom and damp sphagnum moss (B&Q, UK) filled in the lid to maintain a high humidity. Tubes were then placed horizontally on sticky tape in plastic boxes before incubation at 23°C. For ovipositing *Culicoides*, female midges were collected by allowing feeding on authors’ forearms from the Loch Lomond site (May 2018). When midges were observed to be replete (approximately after 5 min), or the insect released mouth parts from the skin, they were aspirated and stored in a plastic container at ambient temperature (13 to 17°C) until the end of the collection session.

Oviposition containers (Fig. S3) were assembled based on previous studies undertaken by Carpenter (67). Briefly, approximately 50 *Culicoides* midges were transferred to containers consisting of cylindrical pill boxes (Watkins and Doncaster, UK; 64 mm diameter, 60 mm depth), with cotton wool soaked in 10% (wt/vol) sucrose solution (replaced every 2 days) placed on top of a fine net meshing which covered the tops of the pillboxes. For ovipositing areas, 50-ml Falcon tube lids were filled with damp sphagnum moss by soaking in 1% nipagin dissolved in distilled water and squeezing until drops could be counted, before being placed on top of damp filter paper. This was secured by cutting a cylindrical hole in the bottom of the pill box. These were then transferred to 19 × 12 × 8 cm plastic boxes containing a 50-ml beaker of saturated sodium sulfate (Na_2_SO_4_), which has previously been described to maintain humidity at >90% when between 20 and 25°C (68). These were then transported to a laboratory in Liverpool before the plastic boxes were placed in an incubator where they were maintained at 23°C with a photoperiod of 12 light:12 dark hours.

### Larval rearing.

Eggs oviposited onto the sphagnum moss substrate were picked individually with a fine paintbrush or the damp edge of a sharpened tungsten needle and placed onto 0.5% agar dishes (100 to 180 eggs per dish; *n* = 3 dishes) and spaced evenly apart. *Culicoides impunctatus* identification was confirmed by the distinctive brown heads of larvae (69), as well as identification of ovipositing adults in pill boxes. Larvae were fed daily on banana worms (Panagrellus nepenthicola), which were cultured using the manufacturer’s instructions (Ron’s Worms, UK) before a fine paintbrush was used to place the nematodes in 2 to 3 ml of deionized water and spread evenly across each agar dish. Immature midges were stored under the same temperature and photoperiods as adults previously mentioned. Larval instars were designated by head capsule length measurements as described by Kettle and Lawson (69).

### Dissections of adults.

Post-blood feeding in the wild, *Culicoides* midges were processed after carefully timed transportation to a laboratory in Liverpool. Individuals were sacrificed at various time points post-blood feeding (PBF); 0 h (non-blood fed), 12 h PBF, and 120 h PBF. First, *Culicoides* midges were chilled in the freezer for immobilization before being placed in a drop of phosphate-buffered saline (PBS) on a petri dish. Midges were then killed by piercing the thorax with a sharp tungsten needle before being confirmed as *C. impunctatus*. Ovaries and other tissues were then exposed through dissection under a stereoscopic microscope. Time points for sacrifice were informed by Carpenter (67), which identified developmental stages of forming eggs as a function of time after blood feeding. Stages were confirmed by using a system developed by Linley (70) that was subsequently modified by Campbell and Kettle (71) as follows: stage 1, no observation of yolk within the oocyte; stage 2, yolk can be identified within the oocyte; stage 3, yolk proteins occupy up to three-quarters of the oocyte; stage 4, the oocyte is elongated and no longer oval, resembling the mature egg; and stage 5, egg fully mature, with chorion visible.

### Tissue preparation and fluorescence *in situ* hybridization.

Tissues examined included eggs of different developmental stages, Malpighian tubules, midgut, foregut, hindgut, fat body, testes, and salivary glands. Additionally, crushed spermathecae were prepared for visualization of the spermatophore and spermatids contained within. To this end, spermathecae were suspended in phosphate-buffered saline and allowed to dry before pressing a coverslip over the slide to break open the tissue.

For fluorescence *in situ* hybridization (FISH) imaging, the above tissues were fixed directly on poly-l-lysine-covered slides for 1 h in Carnoy’s solution (chloroform:ethanol:glacial acetic acid, 6:3:1) and tissues cleared by treating with 6% H_2_O_2_ in ethanol for 2 h. Two prehybridization washes were undertaken using wash buffer (20 mM Tris-HCl, pH 8.0, 50 mM NaCl, 0.01% sodium dodecyl sulfate, 5 mM EDTA). Hybridization was performed overnight in hybridization buffer (20 mM Tris-HCl, pH 8.0, 90 mM NaCl, 0.01% sodium dodecyl sulfate, 30% formamide) containing 10 pmol/ml of the *Rickettsia*-specific probe (5′-CCATCATCCCCTACTACA-[ATTO 633]-3′) adapted from Perotti et al. (24), which was checked for specificity against the Torix 16S gene of Culicoides newsteadi (MWZE00000000). After hybridization, the samples were thoroughly washed twice in wash buffer and slide mounted in Vectashield with DAPI (Vector Laboratories) and viewed under a Zeiss LSM 880 BioAFM confocal microscope. *Rickettsia*-free midges (Culicoides nubeculosus; Pirbright Institute, UK) were used as negative controls. For each tissue, at least 5 specimens were viewed under the microscope to confirm reproducibility (except the single adult male available for analysis). Optical sections (0.7 μm thick) were prepared from each specimen to create a Z-stack image to be processed in ImageJ. All FISH imaging equipment and technical assistance were provided by the Liverpool Centre for Cell Imaging (University of Liverpool, UK).

### Transmission electron microscopy.

As host-seeking *Culicoides* midges mate prior to blood feeding, it was possible to examine spermatids from female spermathecae. Ovaries and spermathecae were prepared for transmission electron microscopy (TEM) as follows. Tissues were dissected into 2% (wt/vol) paraformaldehyde + 2.5% (wt/vol) glutaraldehyde in 0.1 M phosphate buffer (pH 7.4). Fixative was then changed for 2.5% (wt/vol) glutaraldehyde in 0.1 M phosphate buffer (pH 7.4). Heavy metal staining consisted of 2% (wt/vol) OsO_4_ in deionized distilled water (ddH_2_O), followed by 1% (wt/vol) tannic acid in ddH_2_O and then 1% (wt/vol) aqueous uranyl acetate. To prevent precipitation artifacts, the tissue was washed copiously with ddH_2_O between each staining step. Fixation and staining steps were performed in a Pelco BiowavePro (Ted Pella Inc., Redding, CA, USA) at 100W 20 Hg, for 3 min and 1 min, respectively. Dehydration was in a graded ethanol series before filtration and embedding in medium premix resin (TAAB, Reading, UK). For TEM, 70- to 74-nm serial sections were cut using a UC6 ultra microtome (Leica Microsystems, Wetzlar, Germany) and collected on Formvar (0.25% [wt/vol] in chloroform; TAAB, Reading, UK) coated Gilder 200 mesh copper grids (GG017/C, TAAB, Reading, UK). Images were acquired on a 120 kV Tecnai G2 Spirit BioTWIN (FEI, Hillsboro, OR, USA) using a MegaView III camera and analySIS software (Olympus, Germany).

## Supplementary Material

Supplemental file 1
