# Supplementary material for: Tissue Tropisms and Transstadial Transmission of a Rickettsia Endosymbiont in the Highland Midge, Culicoides impunctatus (Diptera: Ceratopogonidae)
Source: Appl Environ Microbiol. 2020 Oct 1;86(20):e01492-20. doi: 10.1128/AEM.01492-20 (PMC7531967; doi:10.1128/AEM.01492-20)
Supplement: Supplemental file 1 [file AEM.01492-20-s0001.pdf]

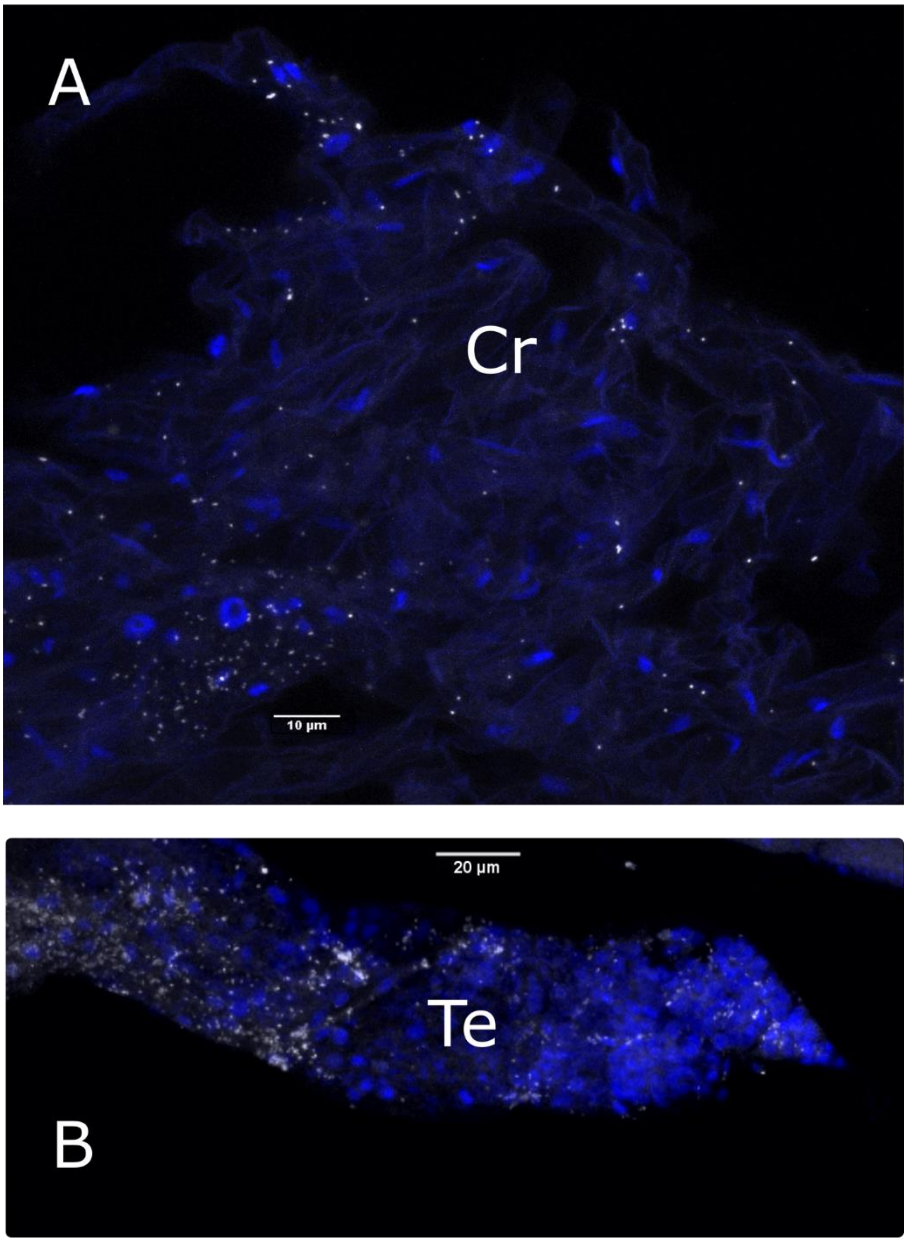

Fig S1. *Rickettsia* infections of **A)** crop (Cr) and **B)** testes (Te). *Rickettsia*-specific probe = white; DAPI-staining = blue.

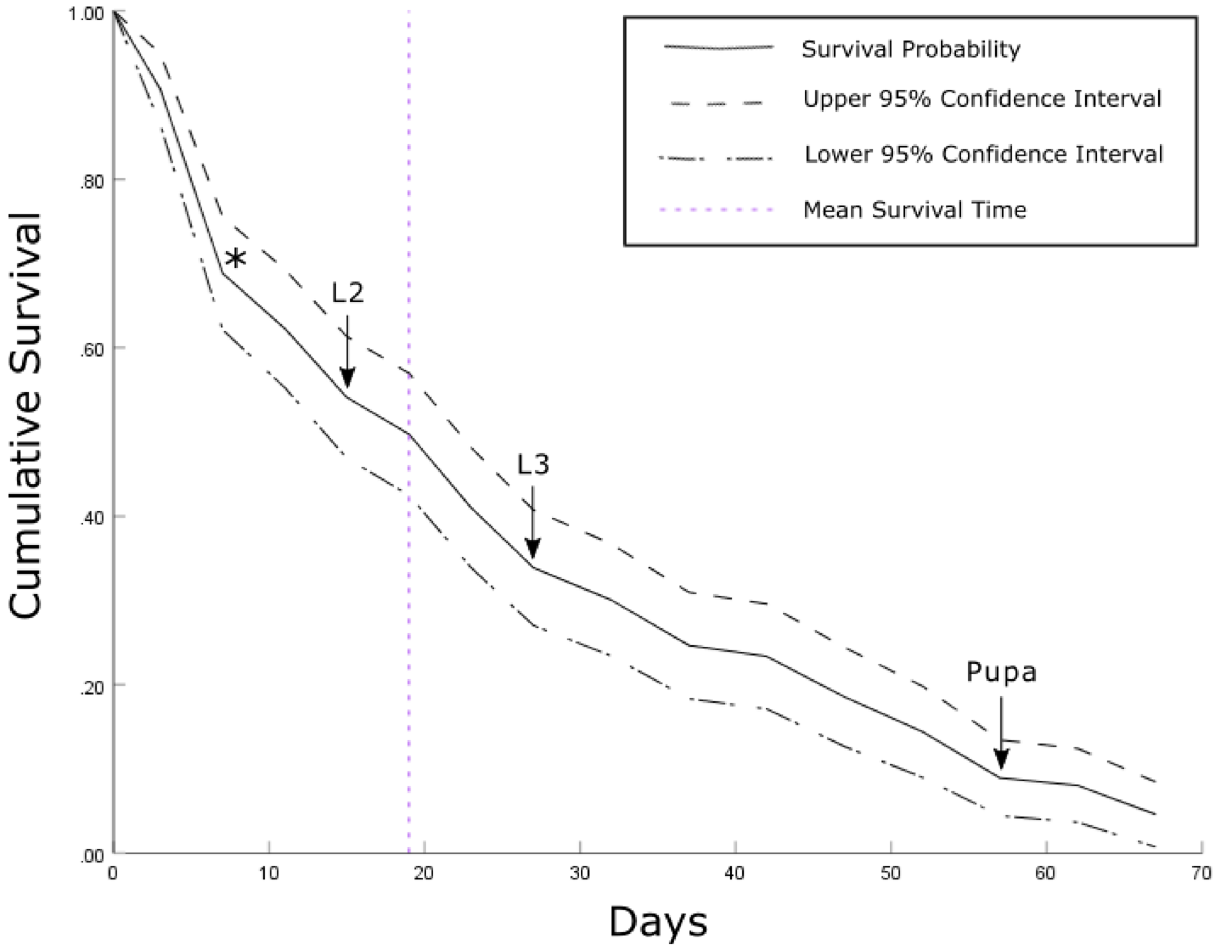

Fig S2. Kaplan-Meier survival curve monitoring cumulative survival of *C. impunctatus* larvae (n=183) over time. Arrows demonstrate the first appearance of different instars. The asterisk is the point at which cannibalism ceased to be observed in L1s. Development into pupae (n=5) took a minimum of 56 days and the mean survival time of larvae was 19 days. **NB**: The burrowing behaviour of mature larvae made for difficult retrieval and head measurements, meaning a formal identification of the first appearance of L4 instars was not achieved.

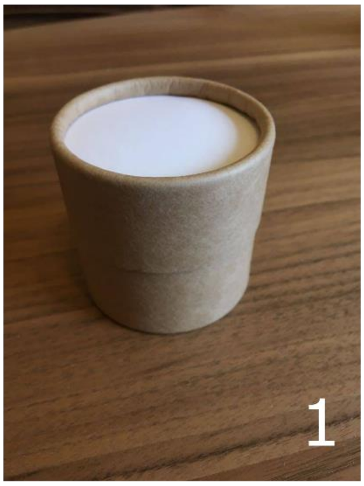

1. Start with a cardboard cylindrical pill box (64 mm diameter x 60mm depth)

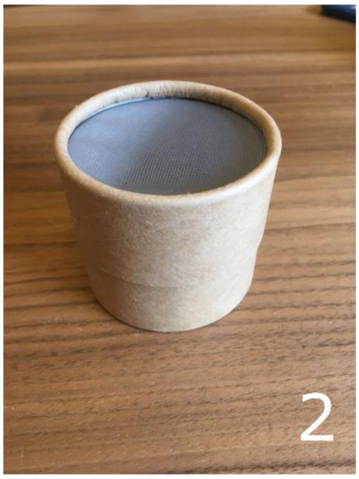

2. Remove top of lid and replace with fine mesh

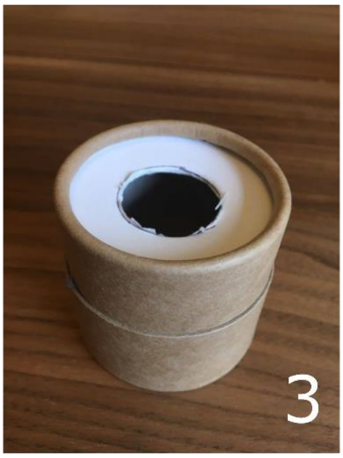

3. Cut a 3cm diameter hole in bottom of container

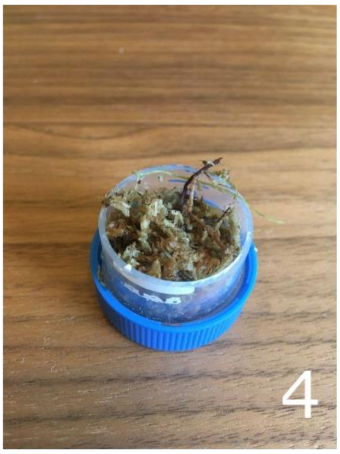

4. Cut the lid of a 50 ml falcon tube and fill with damp sphagnum moss

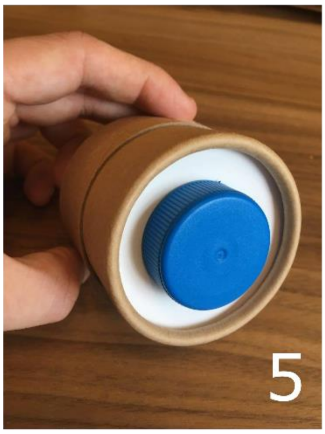

5. After transferring midges into container through the cut hole in step 3, attach falcon lid with moss onto bottom of container

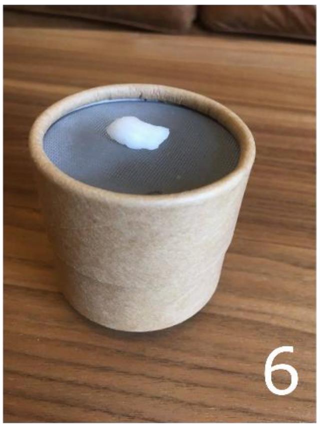

6. Soak a piece of cotton wool in 10% w/v sucrose and place on fine mesh at top of container

Fig S3. Instructions on assembling oviposition containers
